# Modulating the ESIPT Mechanism and Luminescence Characteristics of Two Reversible Fluorescent Probes by Solvent Polarity: A Novel Perspective

**DOI:** 10.3390/molecules29071629

**Published:** 2024-04-05

**Authors:** Yang Wang, Hongyan Mu, Yuhang Sun, Jiaan Gao, Xiaodong Zhu, Hui Li

**Affiliations:** Jilin Key Laboratory of Solid-State Laser Technology and Application, School of Physics, Changchun University of Science and Technology, Changchun 130022, China; wy318321@163.com (Y.W.); xuemuafm@163.com (H.M.); 13136853259@163.com (Y.S.); gja13622147194@163.com (J.G.); zhu1013288433@163.com (X.Z.)

**Keywords:** intramolecular hydrogen bond, intramolecular charge transfer, excited-state intramolecular proton transfer

## Abstract

As reversible fluorescent probes, HTP-1 and HTP-2 have favourable applications for the detection of Zn^2+^ and H_2_S. Herein, the impact of solvent on the excited-state intramolecular proton transfer (ESIPT) of HTP-1 and HTP-2 was comprehensively investigated. The obtained geometric parameters and infrared (IR) vibrational analysis associated with the intramolecular hydrogen bond (IHB) indicated that the strength of IHB for HTP-1 was weakened in the excited state. Moreover, structural torsion and almost no ICT behaviour indicated that the ESIPT process did not occur in HTP-1. Nevertheless, when the 7-nitro-1,2,3-benzoxadiazole (NBD) group replaced the H atom, the IHB strength of HTP-2 was enhanced after photoexcitation, which inhibited the twisting of tetraphenylethylene, thereby opening the ESIPT channel. Notably, hole-electron analysis and frontier molecular orbitals revealed that the charge decoupling effect was the reason for the fluorescence quenching of HTP-2. Furthermore, the potential energy curves (PECs) revealed that HTP-2 was more inclined to the ESIPT process in polar solvents than in nonpolar solvents. With a decrease in solvent polarity, it was more conducive to the ESIPT process. Our study systematically presents the ESIPT process and different detection mechanisms of the two reversible probe molecules regulated by solvent polarity, providing new insights into the design and development of novel fluorescent probes.

## 1. Introduction

Hydrogen bonds (HBs) are chemical bonds formed by interactions between hydrogen atoms and atoms with higher electronegativity (such as nitrogen, oxygen or fluorine) and are among the most important weak interactions within and between molecules [1,2,3,4,5,6]. Owing to their specific saturation and directivity [7,8,9,10], HBs play an extremely vital role in the fields of chemistry, physics and biology [11,12,13,14,15,16]. Excited-state intramolecular proton transfer (ESIPT) is a photophysical process that promotes the interconversion isomerisation between enol and keto in molecules under photoexcitation [17,18,19,20,21]. Because of the significant Stokes displacement emission of the ESIPT process [22,23], it has been extensively used in organic luminescent materials, fluorescent probes, biosensors, molecular light switches and other fields [24,25,26,27,28]. As early as the 1950s, Weller et al. demonstrated that methyl salicylate had ESIPT characteristics and proposed a mechanism of molecular isomerisation leading to double fluorescence [29]. Subsequently, more and more researchers begun to explore the reaction mechanism of ESIPT from both experimental and theoretical levels [30,31].

**Scheme 1:**
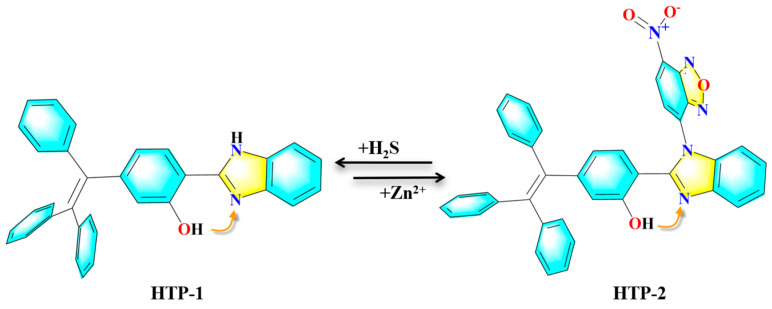
Configurations and detection mechanism of HTP-1 and HTP-2.

In the past few years, 2-(2-hydroxyphenyl) benzoazole (HBX) derivatives had been widely studied as ESIPT molecules with high research value as organic luminescent and fluorescent probe materials [32,33,34,35]. Many previous studies have shown that solvent polarity has a vital effect on the ESIPT process of HBX derivatives in the excited state [36,37,38]. Xiao et al. theoretically studied the ESIPT process and the photophysical mechanism of 2-benzo [b] thiphen-3-yl-3-hydroxy -6-methoxy -chroman-4-one (CHBT) in different solvents. It was confirmed that the ESIPT reaction for CHBT in nonpolar solvents was easier [39]. Du et al. discussed the ESIPT reaction of 6-amino-2-(2′-hydroxyphenyl) benzoxazole (6A-HBO) in different solvents. It was verified that the ESIPT reaction was more prone to occur as the solvent polarity increased [40]. Zhao et al. investigated the excited-state intramolecular double proton transfer (ESDPT) reaction of 2-bis(benzothia-zolyl) naphthalene-diol (2-BBTND) in different polar solvents. It was found that strongly polar solvents promoted the stepwise ESDPT process of 2-BBTND [41]. Recently, Yi et al. designed and synthesised the HBX derivatives 2-(1H-benzo [d] imidazole-2-yl)-5-(1,2,2-triphenylvinyl)-phenol (HTP-1) and 2-(1-(7-nitrobenzo[c] [1,2,5] oxadiazol-4-yl)-1H-benzo [d]imidazol-2-yl)-5-(1,2,2-triphenylvinyl) phenol (HTP-2) [42]. HTP-1 and HTP-2 can detect Zn^2+^ and H_2_S, respectively. HTP-1 exhibits obvious blue fluorescence. When HTP-1 detects Zn^2+^, the combination of HTP-1 and Zn^2+^ significantly enhances the fluorescence, resulting in the production of HTP-2. Pure HTP-2 has no fluorescence, but it releases HTP-1 when detecting H_2_S and exhibits blue fluorescence. The structural difference between HTP-1 and HTP-2 is that the H atom in HTP-1 is replaced by the 7-nitro-1,2,3-benzoxadiazole (NBD), which is correspondingly converted to HTP-2 (Scheme 1). However, the underlying reason for the different luminescence mechanisms of these two molecules remained unclear. It cannot be ignored that there are flexible rotating tetraphenylethylene and ESIPT sites in both molecules. The corresponding photophysical characteristics and luminescence mechanism are worth exploring. In addition, the authors speculate that solvent polarity may further alter the photophysical properties of the molecules [42]. Therefore, it is imperative to promptly investigate the internal mechanism of the excited state for HTP-1 and HTP-2 by solvent polarity. This will theoretically validate the detection mechanism of Zn^2+^ and H_2_S using the two probes.

This study comprehensively investigated the impact of solvent polarity on the ESIPT process of HTP-1 and HTP-2. We calculated the geometric parameters and infrared (IR) vibrational spectra which were associated with the HB interactions. In addition, we simulated the absorption and fluorescence spectra for HTP-1 and HTP-2. According to hole-electron analysis and frontier molecular orbitals (FMOs) diagrams, the internal causes of the different luminescence mechanisms of the two molecules were determined. To provide a more intuitive description for the different ESIPT processes of the two molecules, we constructed potential energy curves (PECs) of four different solvents in the S_0_ and S_1_ states.

## 2. Results and Discussion

### 2.1. Geometric Parameters

**Figure 1:**
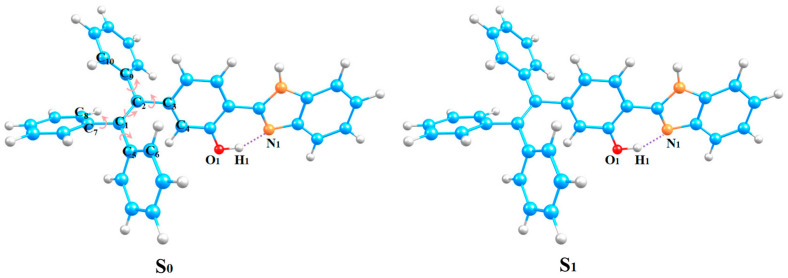
Optimized geometries of HTP-1 using the MN15/6-31G (d, p) method in S_0_ and S_1_ states; blue atom: C, orange atom: N, red atom: O, white atoms: H.

**Figure 2:**
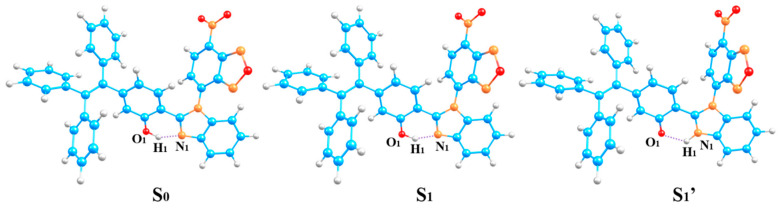
Optimized geometries of HTP-2 using the MN15/6-31G (d, p) method in S_0_, S_1_ and S_1_′ states; blue atom: C, orange atom: N, red atom: O, white atoms: H.

The configurations for HTP-1 and HTP-2 in different electronic excited states were optimized at the theoretical level of MN15/6-31G (d, p). In the excited state, we labelled the E* structure as the S_1_ state and the K* structure as the S_1_ state. The atoms involved were numerically labelled (see Figure 1 and Figure 2), and the calculated geometric parameters for the four solvents (ACN, ACE, DCM and n-Hexane) are recorded in Table 1 and Table S1.

For HTP-1, from the S_0_ to the S_1_ state, the O_1_-H_1_ bond lengths were almost unchanged in four solvents. The N_1_...H_1_ bond lengths from the S_0_ to the S_1_ state elongated by 0.002 Å (ACN), 0.003 Å (ACE), 0.003 Å (DCM) and 0.001 Å (n-Hexane), respectively (see Figure 1). This indicated that the IHBs (O_1_-H_1_...N_1_) did not change significantly in the S_1_ state. Remarkably, after photoexcitation, HTP-1 underwent torsion at multiple positions in different solvents (see Table S1). The dihedral angle α (C_5_-C_1_-C_2_-C_3_) had the most severe torsion. It varied from 12.35° (ACN), 12.3° (ACE), 12.32° (DCM) and 12.24° (n-Hexane) in the S_0_ state to 58.67° (ACN), 53.66° (ACE), 52.75° (DCM) and 50.21° (n-Hexane) in the S_1_ state, which had changed 46.32° (ACN), 41.32° (ACE), 40.43° (DCM) and 37.97° (n-Hexane), respectively. This indicated that the structure of HTP-1 was distorted after photoexcitation, and the degree of torsion was more intense with increasing solvent polarity.

For HTP-2 (Figure 2), in the S_0_ state, the O_1_-H_1_ bond lengths were 0.990 Å, 0.990 Å, 0.989 Å and 0.988 Å in four solvents (ACN, ACE, DCM, n-Hexane), respectively. After photoexcitation, the O_1_-H_1_ bond lengths elongated to 0.999 Å, 1.000 Å, 1.000 Å and 1.003 Å, respectively. Analogously, the N_1_-H_1_ bond lengths were 1.775 Å (ACN), 1.776 Å (ACE), 1.778 Å (DCM) and 1.789 Å (n-Hexane) in the S_0_ state, respectively. In the S_1_ state, the N_1_-H_1_ bond lengths shrunk to 1.687 Å (ACN), 1.685 Å (ACE), 1.682 Å (DCM) and 1.660 Å (n-Hexane). In addition, after photoexcitation, the bond angle (O_1_-H_1_...N_1_) increased in four solvents. The above analysis indicated that the IHBs (O_1_-H_1_...N_1_) were significantly strengthened in the S_1_ state. This facilitated the ESIPT process of HTP-2. The strength of the IHBs increased with decreasing solvent polarity in the excited state. Especially, the degree of IHB strengthening (in DCM solvent) was the most obvious in polar solvents. In addition, we obtained the tautomer configuration (S_1_′ state). In the S_1_′ state, a new IHB (N_1_-H_1_...O_1_) was formed, the O_1_-H_1_ bond length was 1.881 Å. In contrast to that in HTP-1, the introduction of the NBD group replaced the H atom to suppress the structural torsion in HTP-2, causing the molecular structure to tend to be flat.

### 2.2. Potential Energy Curves (PECs)

**Figure 3:**
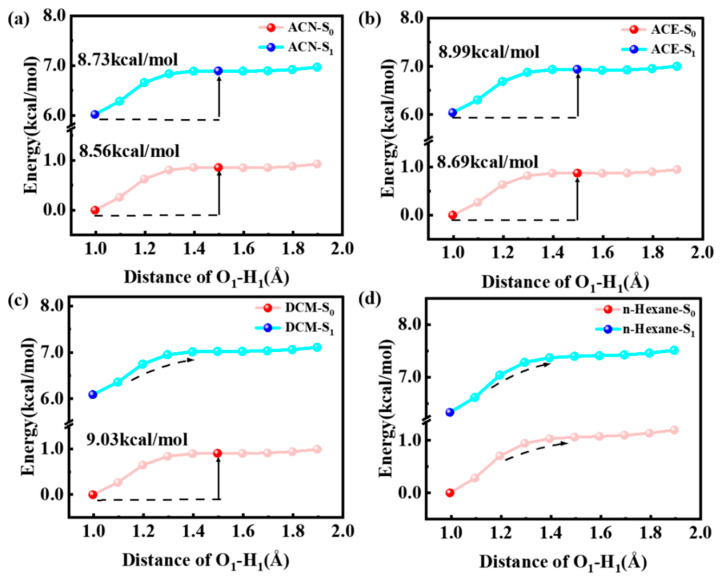
Calculated S_0_- and S_1_-stated potential energy curves (PECs) of HTP-1 in (**a**) ACN, (**b**) ACE, (**c**) DCM and (**d**) n-Hexane solvents.

**Figure 4:**
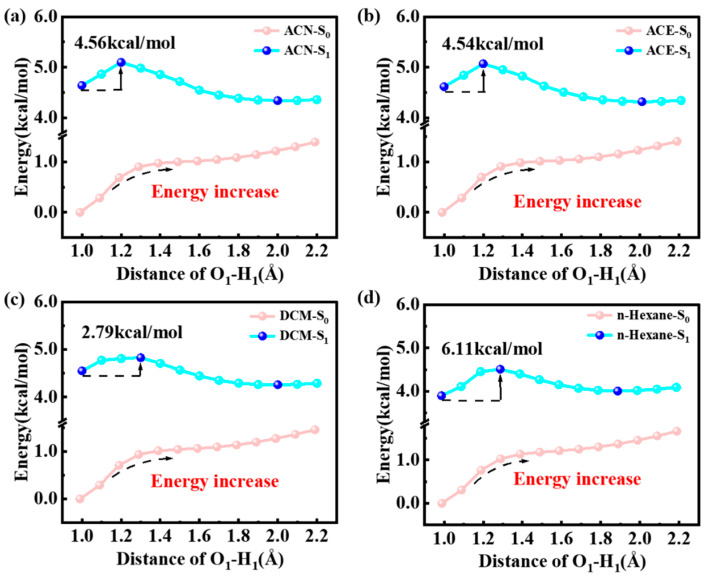
Calculated S_0_- and S_1_-stated potential energy curves (PECs) of HTP-2 in (**a**) ACN, (**b**) ACE, (**c**) DCM and (**d**) n-Hexane solvents.

To explore the degree of difficulty of the ESIPT reaction for HTP-1 and HTP-2, we plotted the S_0_- and S_1_-stated PECs for HTP-1 and HTP-2 in four solvents by fixing the O_1_-H_1_ bond length. As shown in Figure 3, the high barrier and the consistently rising energy of HTP-1 in four solvents made the ESIPT process impracticable in the S_1_ state, indicating that the enol^*^ configuration was more stable. In the S_0_ state, the energy of HTP-2 gradually increased with the extension of the O_1_-H_1_ bond length (see Figure 4). This indicated that the ESIPT reaction was not feasible in the S_0_ state. In the S_1_ state, the energy barriers were 4.56 kcal/mol (ACN), 4.54 kcal/mol (ACE), 2.79 kcal/mol (DCM) and 6.11 kcal/mol (n-Hexane). This result indicated that the ESIPT process tended to occur after photoexcitation. The energy barrier gradually increased with increasing solvent polarity in polar solvents (ACN, ACE and DCM), which was consistent with the order of HB strength. Whereas the energy barrier in nonpolar solvent (n-Hexane) was the highest. This result indicated that the ESIPT process of HTP-2 was more prone to occur in low-polarity solvents.

### 2.3. Infrared (IR) Spectra Analysis

**Figure 5:**
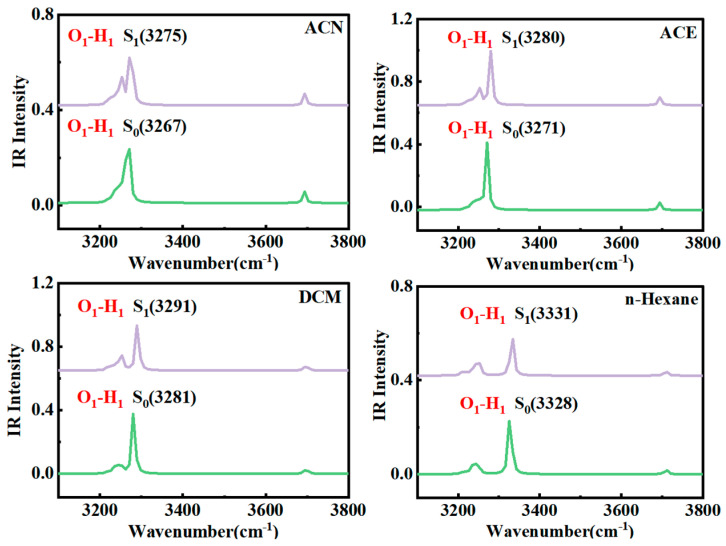
Infrared (IR) vibrational spectra of O_1_–H_1_ and N_1_-H_1_ bonds for HTP-1 in the S_0_ and S_1_ states.

**Figure 6:**
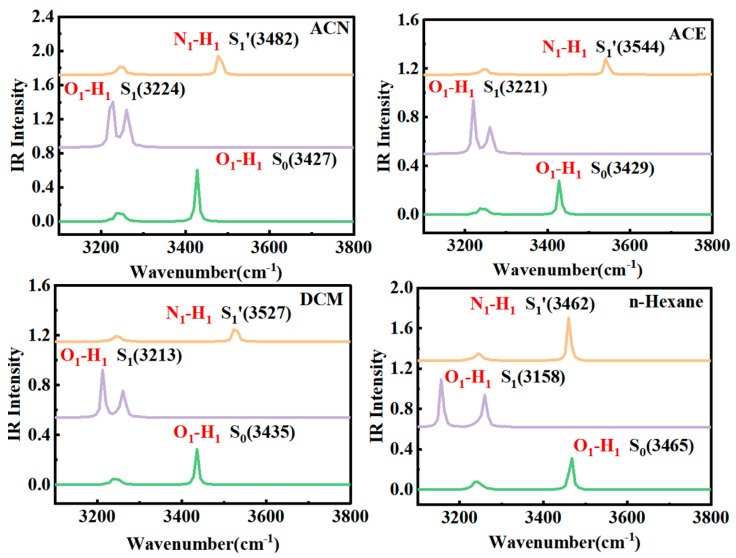
Infrared (IR) vibrational spectra of O_1_–H_1_ and N_1_–H_1_ bonds for HTP-2 in S_0_, S_1_ and S_1_′ states.

Infrared vibrational frequency is a vital indicator to analyse the strength of IHBs [43,44,45,46]. Figure 5 and Figure 6 show the IR vibrational frequencies of O_1_-H_1_ and N_1_-H_1_ for HTP-1 and HTP-2 in four solvents.

For HTP-1, the O_1_-H_1_ stretching modes from the S_0_ to the S_1_ state were blue-shifted in four solvents, resulting in the IHB intensity being weakened in the S_1_ state. The result illustrated that the ESIPT process of HTP-1 was not facile to occur. For HTP-2, the O_1_-H_1_ stretching modes were 3427 cm^−1^ (ACN), 3429 cm^−1^ (ACE), 3435 cm^−1^ (DCM) and 3465 cm^−1^ (n-Hexane) in the S_0_ state, which moved to 3224 cm^−1^, 3221 cm^−1^, 3213 cm^−1^ and 3158 cm^−1^ in the S_1_ state, respectively. Consistent with the geometric analysis, the red shifts in ACN (203 cm^−1^), ACE (208 cm^−1^) and DCM (222 cm^−1^) increased with decreasing solvent polarity. The result indicated that the order of the IHB strength was DCM > ACE > ACN. In addition, the new stretching vibrational mode N_1_-H_1_ was found to replace the original O_1_-H_1_ mode in the S_1_′ state, which situated at 3482 cm^−1^ (ACN), 3544 cm^−1^ (ACE), 3527 cm^−1^ (DCM) and 3462 cm^−1^ (n-Hexane), respectively. Through the above analysis, we found that the IHB strength of HTP-2 was enhanced in four solvents. These results were consistent with the above molecular configuration analysis.

### 2.4. Frontier Molecular Orbitals (FMOs)

**Figure 7:**
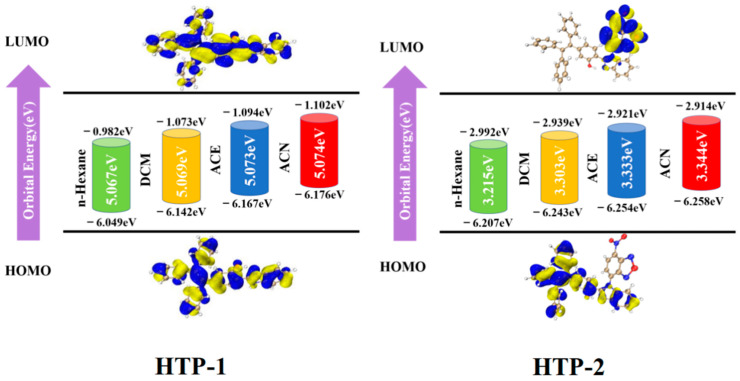
Computational the frontier molecular orbitals (FMOs) and HOMO-LUMO gaps (E_gap_) in HTP-1 and HTP-2.

FMOs are an efficient approach for acquiring changes in molecular charge distribution before and after photoexcitation [47,48]. Figure 7 visually shows the electron density distribution of HTP-1 and HTP-2 at the highest occupied molecular orbital (HOMO) and the lowest unoccupied molecular orbital (LUMO). For HTP-1, the small variation in the charge distribution from HOMO to LUMO indicated that there was almost no ICT process in HTP-1. This finding provided evidence that HTP-1 did not undergo the ESIPT process. For HTP-2, it was obvious that the electron density near the proton donor (O_1_) decreased, whereas the electron density near the acceptor (N_1_) increased during photoexcitation. This facilitated the binding of the N_1_ atom to the H_1_ proton, thus advancing the ESIPT process of HTP-2. Notably, in HTP-2, the degree of overlap for the charge distributions in the two orbitals (HOMO and LUMO) was close to zero, which was the most fundamental reason of the fluorescence quenching in HTP-2. Moreover, the HOMO-LUMO gaps (E_gap_) in different solvents was also calculated. As is well known, the energy gap is inversely proportional to the activity of the chemical reaction [49,50,51,52]. It was not difficult to find that HTP-2 had higher chemical reactivity than HTP-1, and the E_gap_ of HTP-2 increased with enhancing solvent polarity. In addition, to quantificationally study the redistribution of electron density around the IHBs during photoexcitation, we calculated the natural bond orbital (NBO) layout of HTP-1 and HTP-2. For HTP-1, before and after photoexcitation, the charge distribution on the O_1_ and N_1_ atoms was almost unchanged (in Table 2). For HTP-2, the charge on the O_1_ atom hardly changed before and after photoexcitation. However, the charge on the N_1_ atom in ACN, ACE, DCM and n-Hexane solvents were −0.547, −0.546, −0.544 and −0.536 in the S_0_ state, which increased to −0.556, −0.556, −0.555 and −0.553 in the S_1_ state. The increase in charge on the N_1_ atom enhanced its ability to attract protons (H_1_), which could promote the proton transfer in the excited state. The results indicated that ICT behaviour occurred and facilitated the ESIPT tendency in HTP-2.

### 2.5. Electronic Spectra and Hole-Electron Analysis

To preferably comprehend the photophysical properties of HTP-1 and HTP-2 in four solvents, we simulated the absorption and fluorescence spectra using the MN15/6-31G (d, p) method. The main photophysical parameters of HTP-1 and HTP-2 are recorded in Table 3 and Table 4, respectively.

**Figure 8:**
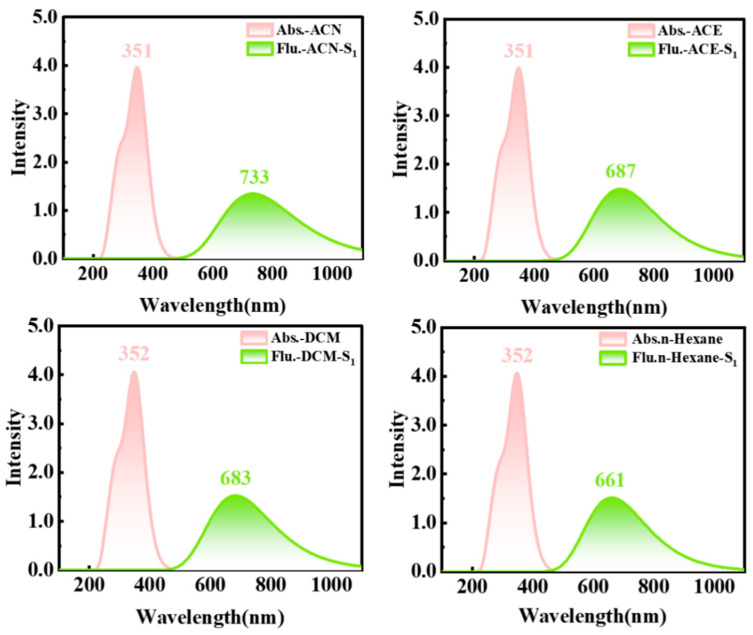
Simulated absorption and emission spectra of HTP-1 in ACN, ACE, DCM and n-Hexane solvents.

**Figure 9:**
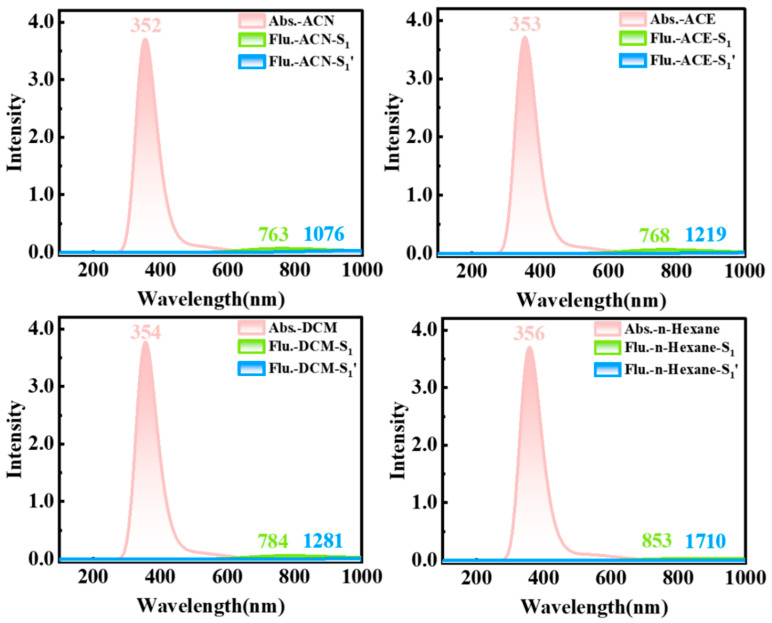
Simulated absorption and emission spectra of HTP-2 in ACN, ACE, DCM and n-Hexane solvents.

For HTP-1 (see Figure 8), the simulated absorption peak (351 nm) in ACN solvent was in agreement with the experimental data (348 nm). This indicated that the adopted MN15/6-31G (d, p) method was extremely reliable for further investigation of photophysical properties in other solvents. In addition, the absorption peaks were at 351 nm (ACE), 352 nm (DCM) and 352 nm (n-Hexane), implying that solvent polarity had little effect on them. The fluorescence peaks of the enol^*^ form in ACN, ACE, DCM and n-Hexane corresponded to 733 nm, 687 nm, 683 nm and 661 nm, respectively. The result distinctly implied that the short wavelength Stokes shift was 309–382 nm and that the fluorescence of the HTP-1 came from enol^*^ form. Meanwhile, for HTP-2 (see Figure 9), the absorption peaks in the four solvents were located at 352 nm (ACN), 352 nm (ACE), 354 nm (DCM) and 356 nm (n-Hexane). Moreover, the fluorescence of the enol^*^ and keto^*^ states for HTP-2 was quenched, which was consistent with the fact that no fluorescence was observed in the experiment [42].

**Figure 10:**
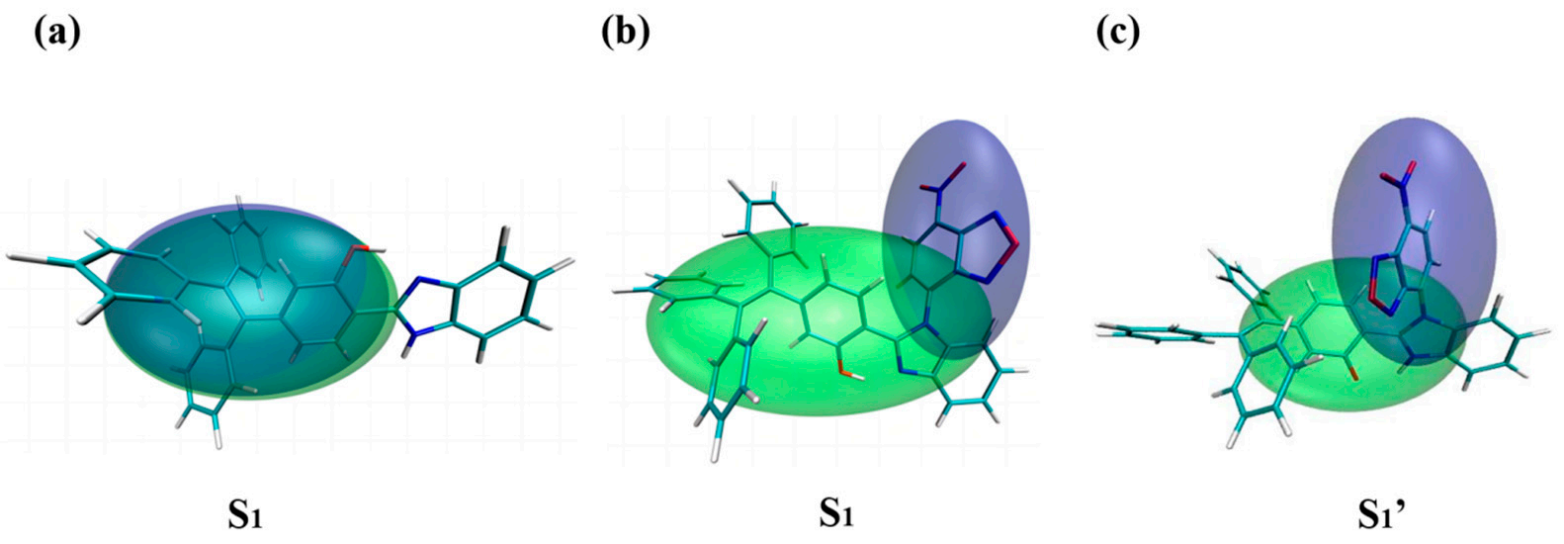
Hole-electron analysis (green and blue regions represent electrons and holes, respectively) in ACN. (**a**) S_1_ form of HTP-1, (**b**) S_1_ form of HTP-2 and (**c**) S_1_′ form of HTP-2.

To explain this phenomenon, we performed the hole-electron analysis shown in Figure 10. The correlative indicators depicting the S_1_ and S_1_′ forms of HTP-1 and HTP-2 in four solvents were included in Table 5. The index D symbolizes the distance between the hole centroid and the electron centroid. The index D symbolizes the distance between holes centroid and electrons centroid, H reflects the overall average distribution width for holes and electrons, Sr describes the extent to which holes and electrons overlap and t represents the degree of separation between holes and electrons. In general, the larger the D index was, the lower the Sr index was and the more positive the t index was, which led to a more adequate separation of electrons and holes. Obviously, both D and t indices of HTP-2 were large, whereas the Sr indices were minimal (between 0.1 and 0.2). The above data indicated that the overlap of electrons and holes was small, which enhanced the ICT process of HTP-2. However, compared with that of HTP-2, the t index of HTP-1 was negative (−1.967). This implied that there was little separation between the distributions of holes and electrons. The hole-electron analysis also verified that HTP-1 did not undergo the ICT process and that the extremely small overlap of holes and electrons was the reason for the fluorescence quenching of HTP-2.

### 2.6. Reduced Density Gradient (RDG) Analysis

To visually differentiate the different interaction types and analyse the influence of solvent polarity on IHB strength [53], we calculated the RDG scatter plots and isosurface plots of HTP-1 and HTP-2 in four solvents. The expression was:

(1) $$RDG(r)=\frac{1|\nabla \rho (r)|}{2{\left(3\pi^{2}\right)}^{1/3}\rho {\left(r\right)}^{4/3}}$$

The second eigenvalue equation of the Hessian matrix was:

(2) $$\Omega (r)=Sign(\lambda_{2}(r))\rho (r)$$

**Figure 11:**
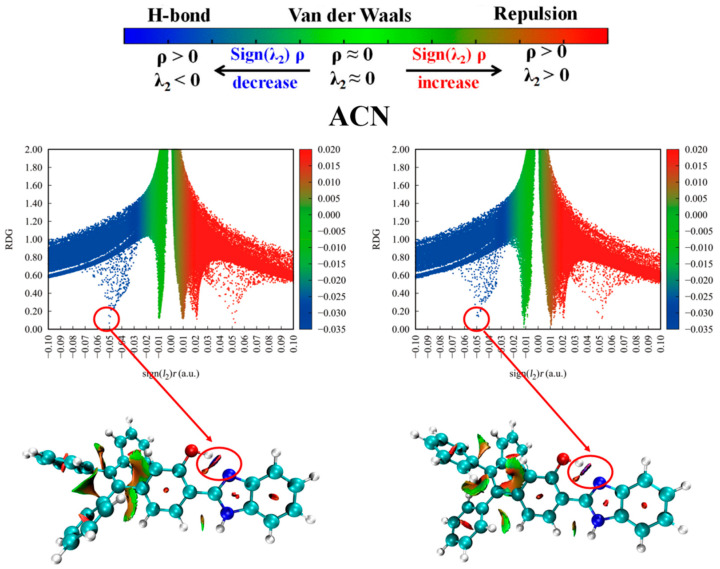
Reduced density gradient (RDG) scatters and isosurfaces for ACN of HTP-1.

**Figure 12:**
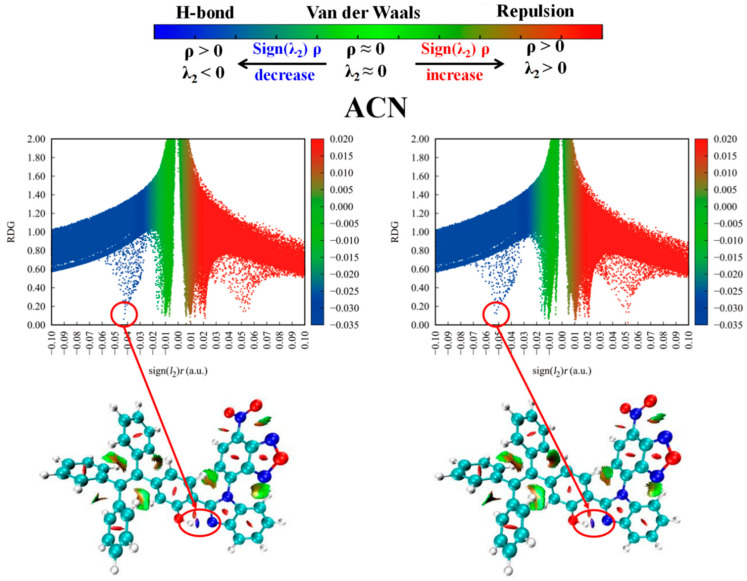
Reduced density gradient (RDG) scatters and isosurfaces for ACN of HTP-2.

As shown in the colour scale at the top of Figure 11 and Figure 12, blue represents HB interactions, and the HB intensity increased gradually with the deepening of the colour. Red represents the spatial effects, and green represents the van der Waals interactions. In the S_0_ state (see Figures S1–S3), the spike peaks of HTP-1 and HTP-2 in four solvents were located at −0.05 and −0.041~−0.43, respectively. The interaction type was ascribed to the HB interaction, corresponding to IHB O_1_-H_1_...N_1_. After photoexcitation, the spike peaks of HTP-1 right-shifted to −0.046~−0.049, indicating that IHBs (O_1_-H_1_...N_1_) were weakened in the excited state. However, the spike peaks of HTP-2 left-shifted to −0.051~−0.056, indicating that IHBs (O_1_-H_1_...N_1_) were enhanced in the excited state. The result was consistent with molecular configuration and IR spectra analysis. The non-covalent interaction analysis confirmed the existence of an HB interaction. Moreover, the strength of IHBs for HTP-1 did not change with the solvent polarity, whereas the IHB strength for HTP-2 in polar solvents followed the order of DCM > ACE > ACN.

## 3. Theoretical Methods

All theoretical calculations were conducted using Gaussian 16 software [54]. Using the density functional theory (DFT) and time-dependent density functional theory (TD-DFT) methods, all geometric structures of HTP-1 and HTP-2 were completely optimized in four solvents (polarity order of solvents: acetonitrile (ACN) > acetone (ACE) > dichloromethane (DCM) > n-Hexane, see Tables S2 and S3). To simulate the experimental surroundings, we used the polarizable continuum model (PCM) of the formal variant of the integral equation (IEFPCM) [55]. In addition, we selected five typical functionals to calculate the absorption spectrum of HTP-1 in ACN solvent. Accordingly, the absorption peak (351 nm) obtained by MN15/6-31G (d, p) was the closest to the experimental data (348 nm) (see Table 6), which proved the dependability of selecting MN15/6-31G (d, p) for further theoretical calculations. To quantitatively analyse the charge distribution around the IHB after photoexcitation, we calculated the natural bond orbital (NBO) charge distribution. In addition, to visually demonstrate the internal mechanism of the ESIPT reaction, we scanned the S_0_- and S_1_ stated PECs of HTP-1 and HTP-2 in ACN, ACE, DCM and n-Hexane solvents. The hole-electron analysis, FMOs and the RDG parameters were performed by the Multiwfn program [56,57,58,59], and VMD 1.9.4 software was used for visualization [60].

## 4. Conclusions

In summary, the influence of solvent polarity on the photophysical mechanism of HTP-1 and HTP-2 was systematically elucidated based on quantum chemistry methods. Through the analysis of the IHB geometric parameters and IR vibrational spectra, it could be inferred that during photoexcitation the IHB strength of HTP-1 weakened. In addition, the FMOs diagram and NBO charge distribution revealed minimal changes in the electron density distribution of HTP-1, indicating that there was almost no ICT process. The structural torsion and lack of ICT behaviour confirmed that HTP-1 did not undergo the ESIPT process. However, when the NBD group replaced the H atom, the IHB strength of HTP-2 was enhanced after photoexcitation, which inhibited the twisting of tetraphenylethylene, thereby opening the ESIPT channel. Furthermore, the hole-electron analysis further proved that the complete non-overlapping charge distribution was the reason for the fluorescence quenching of HTP-2. Finally, by constructing the S_0_- and S_1_-state PECs, we elucidated that the ESIPT process for HTP-2 was more prone to occur in polar solvents than that in nonpolar solvents. It was more beneficial to the ESIPT process with decreasing solvent polarity. We sincerely expected that this work could furnish valuable theoretical guidance for the development of high-performance fluorescent probes and promote the development of fluorescent material applications.

## Figures and Tables

**Table 1:** Optimized geometric parameters of HTP-1 and HTP-2 in different electron states. bond lengths (Å), bond angles and dihedral angles (°).

			O_1_-H_1_	N_1_-H_1_	δ(O_1_-H_1_-N_1_)	C_5_-C_1_-C_2_-C_3_
HTP-1	ACN	S_0_	0.997	1.707	147.79	12.35
		S_1_	0.997	1.709	148.12	58.67
	ACE	S_0_	0.997	1.708	147.74	12.34
		S_1_	0.997	1.711	148.07	53.66
	DCM	S_0_	0.997	1.711	147.63	12.32
		S_1_	0.996	1.714	147.94	52.75
	n-Hexane	S_0_	0.995	1.726	147.08	12.24
		S_1_	0.994	1.727	147.34	50.21
HTP-2	ACN	S_0_	0.990	1.775	145.68	12.56
		S_1_	0.999	1.687	146.60	20.22
		S_1_′	1.881	1.026	126.13	12.63
	ACE	S_0_	0.990	1.776	145.64	12.53
		S_1_	1.000	1.685	146.63	20.24
		S_1_′	2.010	1.022	120.44	9.69
	DCM	S_0_	0.989	1.778	145.54	12.46
		S_1_	1.000	1.682	146.71	20.32
		S_1_′	1.981	1.023	121.90	9.63
	n-Hexane	S_0_	0.988	1.789	145.10	12.23
		S_1_	1.003	1.660	147.13	20.75
		S_1_′	1.886	1.027	126.50	9.90

**Table 2:** Natural bond orbital (NBO) charge distribution over the O_1_ and N_1_ atoms of HTP-1 and HTP-2 in ACN, ACE, DCM and n-Hexane solvents (a.u.).

		O_1_		N_1_	
		S_0_	S_1_	S_0_	S_1_
HTP-1	ACN	−0.727	−0.729	−0.577	−0.576
	ACE	−0.726	−0.728	−0.576	−0.575
	DCM	−0.724	−0.726	−0.574	−0.573
	n-Hexane	−0.712	−0.714	−0.565	−0.564
HTP-2	ACN	−0.721	−0.721	−0.547	−0.556
	ACE	−0.720	−0.720	−0.546	−0.556
	DCM	−0.718	−0.718	−0.544	−0.555
	n-Hexane	−0.709	−0.710	−0.536	−0.553

**Table 3:** Calculated transition properties of HTP-1 in four solvents (corresponding oscillator intensity (f) and component indexes CI (%)).

		Transition	λ_abs_ (nm)	f	Composition	CI (%)
HTP-1	ACN	S_0_→S_1_	350.9	0.9149	H→L	68.84%
		S_0_→S_2_	304.6	0.3238	H-1→L	57.83%
		S_0_→S_3_	282.3	0.2764	H→L+1	54.34%
	ACE	S_0_→S_1_	351.2	0.9190	H→L	68.83%
		S_0_→S_2_	304.9	0.3238	H-1→L	57.93%
		S_0_→S_3_	282.4	0.2784	H→L+1	54.40%
	DCM	S_0_→S_1_	352.3	0.9351	H→L	68.81%
		S_0_→S_2_	306.0	0.3278	H-1→L	58.17%
		S_0_→S_3_	283.0	0.2853	H→L+1	54.43%
	n-Hexane	S_0_→S_1_	352.0	0.9315	H→L	68.77%
		S_0_→S_2_	308.2	0.2853	H-1→L	59.58%
		S_0_→S_3_	283.7	0.3000	H→L+1	56.36%

**Table 4:** Calculated transition properties of HTP-2 in four solvents. (corresponding oscillator intensity (f) and component indexes CI (%)).

		Transition	λ_abs_ (nm)	f	Composition	CI (%)
HTP-2	ACN	S_0_→S_1_	515.6	0.0230	H→L	66.18%
		S_0_→S_2_	413.1	0.0018	H-1→L	65.46%
		S_0_→S_3_	393.2	0.1181	H-3→L	66.90%
		S_0_→S_4_	374.6	0.0008	H-2→L	65.80%
		S_0_→S_5_	352.2	0.8471	H→L+2	65.34%
	ACE	S_0_→S_1_	517.9	0.0230	H→L	66.19%
		S_0_→S_2_	414.7	0.0018	H-1→L	65.51%
		S_0_→S_3_	393.8	0.1186	H-3→L	66.89%
		S_0_→S_4_	375.6	0.0008	H-2→L	65.64%
		S_0_→S_5_	352.5	0.8508	H→L+2	65.52%
	DCM	S_0_→S_1_	523.8	0.0233	H→L	66.21%
		S_0_→S_2_	418.9	0.0017	H-1→L	65.64%
		S_0_→S_3_	395.6	0.1216	H-3→L	66.77%
		S_0_→S_4_	378.0	0.0009	H-2→L	65.16%
		S_0_→S_5_	353.7	0.8664	H→L+2	65.85%
	n-Hexane	S_0_→S_1_	542.7	0.0224	H→L	66.34%
		S_0_→S_2_	432.7	0.0016	H-1→L	66.11%
		S_0_→S_3_	398.3	0.1166	H-3→L	62.45%
		S_0_→S_4_	385.4	0.0025	H-2→L	62.72%
		S_0_→S_5_	355.5	0.7547	H→L+2	61.28%

**Table 5:** Indexed characterization of holes and electrons for HTP-1 and HTP-2 in four solvents (D: the distance between the hole centroid and the electron centroid. Sr: the degree of overlap between the holes and the electrons. H: the overall average distribution width between the holes and the electrons. t: the degree of separation between the holes and the electrons).

			D (Å)	Sr	H (Å)	T (Å)
HTP-1	S_1_	ACN	0.214	0.89852	3.542	−1.967
		ACE	0.182	0.88927	3.569	−1.880
		DCM	0.176	0.88848	3.570	−1.830
		n-Hexane	0.155	0.86627	3.570	−1.689
HTP-2	S_1_	ACN	5.979	0.13088	3.453	3.723
		ACE	5.997	0.12876	3.450	3.749
		DCM	6.042	0.12376	3.439	3.807
		n-Hexane	6.208	0.10119	3.385	4.041
	S_1_′	ACN	5.091	0.13141	2.652	3.511
		ACE	4.682	0.15186	2.601	3.072
		DCM	4.073	0.14849	2.588	3.102
		n-Hexane	4.746	0.12309	2.528	3.192

**Table 6:** Calculated absorption peaks (nm) of HTP-1 using five functionals. (Exp. is the experimental value).

	B3LYP	CAM-B3LYP	M06-2X	MPW1PW91	MN15	Exp.
λ_abs_	374	314	326	364	351	348

## Data Availability

Data are contained within the article and Supplementary Materials.

## Supplementary Materials

The following supporting information can be downloaded at: https://www.mdpi.com/article/10.3390/molecules29071629/s1, Figure S1: Reduced density gradient (RDG) versus X(r) scatter plots in ACE: (a) HTP-1, (b) HTP-2.; Figure S2: Reduced density gradient (RDG) versus X(r) scatter plots in DCM: (a) HTP-1, (b) HTP-2. Figure S3. Reduced density gradient (RDG) versus X(r) scatter plots in n-Hexane: (a) HTP-1, (b) HTP-2. Table S1: Bond length (Å), bond angles and dihedral angles (°) of HTP-1 and HTP-2 in different electron states.; Table S2: Dielectric constant and polarity of four solvents. Table S3: The experimental and theoretical data of absorption and emission spectra of HTP-1 and HTP-2 in four solvents.

## References

[1] Zhou P.W., Han K. (2018). Unraveling the Detailed Mechanism of Excited-State Proton Transfer. Acc. Chem. Res..

[2] Chen R.T., Ren Z.F., Liang Y., Zhang G.H., Dittrich T., Liu R.Z., Liu Y., Zhao Y., Pang S., An H.Y. (2022). Spatiotemporal imaging of charge transfer in photocatalyst particles. Nature.

[3] Kwon J.E., Park S.Y. (2011). Advanced organic optoelectronic materials: Harnessing excited-state intramolecular proton transfer (ESIPT) process. Adv. Mater..

[4] Zhao J.F., Liu C. (2023). Computational Insights into Excited State Intramolecular Double Proton Transfer Behavior Associated with Atomic Electronegativity for Bis(2′-benzothiazolyl) hydroquinone. Molecules.

[5] Wang Y.X., Zhang M., Li W.Z., Wang Y., Zhou P.W. (2023). Theoretical Investigation on the “ON-OFF” Mechanism of a Fluorescent Probe for Thiophenols: Photoinduced Electron Transfer and Intramolecular Charge Transfer. Molecules.

[6] He L., Dong B., Liu Y., Lin W. (2016). Fluorescent chemosensors manipulated by dual/ triple interplaying sensing mechanisms. Chem. Soc. Rev..

[7] Zhao G.J., Northrop B., Han K., Stang P. (2010). The effect of intermolecular hydrogen bonding on the fluorescence of a bimetallic platinum complex. J. Phys. Chem. A.

[8] Zhu L.X., Li Q., Wan Y.F., Guo M.L., Yan L., Yin H., Shi Y. (2023). Short-Range Charge Transfer in DNA Base Triplets: Real-Time Tracking of Coherent Fluctuation Electron Transfer. Molecules.

[9] Li H., Mu H.Y., Xin C., Cai J.X., Yuan B.S., Jin G.Y. (2022). Turning ON/OFF the fluo-rescence of the ESIPT state by changing the hydrogen bond distance and ori-entation in quinoline-pyrazole derivatives. J. Mol. Struct..

[10] Xin X., Zhao Y., Shi W., Zhao G.J., Li Y.Q. (2022). Effects of Twisted Intramolecular Charge Transfer Behavior on Excited-State Intramolecular Proton Transfer Reactions of Methyl Benzoate Derivatives in Water Solution. J. Phys. Chem. A.

[11] Zhao G.J., Yang Y.F., Zhang C.Y., Song Y.Z., Li Y.Q. (2021). The theoretical study of excited-state intramolecular proton transfer of N,N,-bis (salicylidene)-(2-(3″4′-diaminophenyl) benzothiazole). Spectrochim. J. Lumin..

[12] Jiang G.S., Tang Z., Han H.Y., Ding J.X., Zhou P.W. (2021). Effects of Intermolecular Hydrogen Bonding and Solvation on Enol–Keto Tautomerism and Photophysics of Azomethine–BODIPY Dyads. J. Phys. Chem. B.

[13] Mu H.Y., Sun Y.H., Gao J.A., Xin C., Zhao H.F., Jin G.Y., Li H. (2023). Switching the ESIPT and TICT process of DP-HPPI via intermolecular hydrogen bonding. J. Mol. Struct..

[14] Mu H.Y., Li H., Sun C.F., Gao J.A., Yang M., Xin C., Jin G.Y. (2023). Different competition mechanism between ESPT and TICT process regulated by protic and aprotic solvent in DHP. J. Mol. Liq..

[15] Gawrys P., Morawski O., Banasiewicz M., Barboza C.A. (2023). Magnifying the ESIPT process in tris(salicylideneanilines) via the steric effect-a pathway to the molecules with panchromatic fluorescence. Phys. Chem. Chem. Phys..

[16] Zhao G.J., Han K.L. (2007). Early time hydrogen-bonding dynamics of photoexcited coumarin 102 in hydrogen-donating solvents: Theoretical study. J. Phys. Chem. A.

[17] Oftadeh M., Barfarakh Z., Ravari F. (2021). Luminescent excited-state intramolecular proton-transfer dyes based on 4-functionalized 6,6′-dimethyl-3,3′-dihydroxy-2,2′-bipyridine (BP(OH)2-Rs); DFT simulation study. J. Mol. Graph. Model..

[18] Lu T., Chen Q. (2021). Interaction Region Indicator: A Simple Real Space Function Clearly Revealing Both Chemical Bonds and Weak Interactions. Chem.-Methods.

[19] Sun L.C., Chen Y.C., Sun M.T. (2022). Exploring nonemissive excited-state intramolecular proton transfer by plasmon-enhanced hyper-raman scattering and two-photon excitation fluorescence. J. Phys. Chem. C.

[20] Mu H.Y., Li D., Gao J.A., Wang Y., Zhang Y.F., Jin G.Y., Li H. (2023). Achieving the cooperative and stepwise regulation of ESIDPT process in AFBD by introducing different electronic groups. J. Mol. Struct..

[21] Gao J.A., Mu H.Y., Zhen Q., Guan X.Y., Li H. (2023). Accelerating the concerted double proton transfer process of 2,2′-bipyridine-3,3′-diol-5,5′-dicarboxylate acid ethyl ester (BPDC) molecule by centrosymmetric dual intermolecular hydrogen bonds. J. Mol. Struct..

[22] Gao J.A., Zhang Y.F., Mu H.Y., Yang M., Guan X.T., Jin G.Y., Li H. (2023). Paying Comprehensive Attention to the Temperature-Dependent Dual-Channel Excited-State Intramolecular Proton Transfer Mechanism of Fluorescence Ratio Probe BZ-DAM. Int. J. Mol. Sci..

[23] Lou Z.R., Zhou X.Y., Tang Z., Zhou P.W. (2020). Theoretical Insights into the Excited State Decays of a Donor–Acceptor Dyad: Is the Twisted and Rehybridized Intramolecular Charge-Transfer State Involved?. J. Phys. Chem. B.

[24] You J., Cao D., Hu T., Ye Y., Jia X., Li H., Hu X., Dong Y., Ma Y., Wang T. (2021). Novel Norrish type I flavonoid photoinitiator for safe LED light with high activity and low toxicity by inhibiting the ESIPT process. Dyes Pigment..

[25] Sun C.F., Su X., Zhou Q., Shi Y. (2019). Regular tuning of the ESIPT reaction of 3-hydroxychromone-based derivatives by substitution of functional groups. Org. Chem. Front..

[26] Li Q., Zhu L.X., Wan Y., Wan Y.F., Gao J.B., Yin H., Shi Y. (2023). Accelerating ultrafast processes in hydrogen-bonded complexes under pressure. Appl. Phys. Lett..

[27] Li Y.Q., Ma Y.Z., Yang Y.F., Shi W., Lan R.F., Guo Q. (2018). Effects of different substituents of methyl 5-R-salicylates on the excited state intramolecular proton transfer process. Phys. Chem. Chem. Phys..

[28] Yang D.P., Zhao J.F., Yang G., Song N.H., Zheng R., Wang Y.S. (2017). A theoretical study about excited state behaviour for imide compound N-cyclohexyl-3-hydroxyphthalimide and 3,6-dihydroxy-N-cyclohexy lphthalimide. J. Mol. Liq..

[29] Weller A. (1955). Über die Fluoreszenz der Salizylsäure und verwandter Verbindungen. Naturwissenschaften.

[30] Li H., Han J.H., Zhao H.F., Liu X.C., Ma L.N., Sun C.F., Yin H., Shi Y. (2018). Investigation of the Intermolecular Hydrogen Bonding Effects on the Intramolecular Charge Transfer Process of Coumarin 340 in Tetrahydrofuran Solvent. J. Clust. Sci..

[31] Li H., Yin H., Liu X.C., Shi Y., Jin M.X., Ding D.J. (2017). An experimental and theoretical study of solvent hydrogen-bond-donating capacity effects on ultrafast intramolecular charge transfer of LD 490. Spectrochim. Acta Part A Mol. Biomol. Spectrosc..

[32] Santos J.L.F., de Souza G.L.C. (2021). Water hydrogen-bonding effects on the ground and low-lying excited states of dipyridyl isomers. J. Mol. Liq..

[33] Chen L., Fu P.Y., Wang H.P., Pan M. (2021). Excited-state intramolecular proton transfer (ESIPT) for optical sensing in solid state. Adv. Opt. Mater..

[34] Zhai H.S., Zhu M.Y., Jia X.L., Liu Y., Guan T.T., Yang Y.G., Liu Y.F. (2022). Theoretical investigation on the fluorescence properties and ESIPT mechanism of the Al^3+^ ion sensor 1-((2-hydroxynaphthalen-1-yl) methylene) urea (OCN). Spectrochim. Acta Part A Mol. Biomol. Spectrosc..

[35] Kumar G., Singh I., Goel R., Paul K., Luxami V. (2021). Dual-channel ratiometric recognition of Al^3+^ and F ions through an ESIPT-ESICT signalling mechanism. Spectrochim. Acta Part A Mol. Biomol. Spectrosc..

[36] Zhang Q.Q., Yang Y.G., Liu Y.F. (2023). Recognition mechanism of imidazo[1,5-α] pyridine-based fluorescence probe towards thiophenols with multi-mechanisms of PET and ESIPT. J. Photochem. Photobiol. A Chem..

[37] Trannoy V., Le’austic A., Gadan S., Guillot R., Allain C., Clavier G., Mazerat S., Geffffroy B., Yu P. (2021). A highly efficient solution and solid state ESIPT fluorophore and its OLED application. New J. Chem..

[38] Zhang X., Hu S.P., Ma Q., Liao S.H. (2020). Visible light-mediated ring-opening polymerization of lactones based on the excited state acidity of ESPT molecules. Polym. Chem..

[39] Xiao S.S., Lou Z.R., Ji D.B., Zhao J.F. (2021). Understanding solvent polarity dependent excited state behavior and ESIPT mechanism for 2-benzo[b]thiphen-3-yl-3-hydroxy-6-methoxy-chroman-4-one compound. Chem. Phys. Lett..

[40] Du C., Zhou Q., Zhang M.X., Song P., Ma F.C. (2019). Excited-state intramolecular proton transfer of 6-amino-2-(2′-hydroxyphenyl) benzoxazole (6A-HBO) in different solvents. J. Phys. Org. Chem..

[41] Zhao J.F., Yang Y.F., Li L., Zhang H.H. (2023). Theoretical Insights into Excited-State Stepwise Double Proton Transfer Associated with Solvent Polarity for 2-bis(benzothia-zolyl) naphthalene-Diol Compound. Chem. Sel..

[42] Yi S.Q., Liu H.L., Chen Z., Fan C.B., Liu G., Pu S.Z. (2023). Novel fluorescent probes based on NBD-substituted imidazole amino to sequentially detect H_2_S and Zn^2+^. Dyes Pigment..

[43] Shang C.J., Cao Y.J., Shao Z.Q., Sun C.F., Li Y.Z. (2022). Tactfully unveiling the effect of solvent polarity on the ESIPT mechanism and photophysical property of the 3-hydroxylflavone derivative. Spectrochim. Acta Part A Mol. Biomol. Spectrosc..

[44] Yang D.P., Liu W., Kong L.H., Zhang Q.L., Liu Y.F. (2020). Ultrafast excited state intramolecular proton transfer (ESIPT) mechanism for 2,6-bis(benzothiazolyl-2-yl)phenol: A theoretical investigation. Chem. Phys. Lett..

[45] Zhou Q., Du C., Yang L., Dai Y.M., Song P. (2017). Mechanism for the excited-State multiple proton transfer process of dihydroxyanthraqui-none chromophores. J. Phys. Chem. A.

[46] Zhao G.J., Han K.L. (2012). Hydrogen bonding in the electronic excited state. Acc. Chem. Res..

[47] Yang D.P., Yang W.P., Tian Y.S., Zheng R. (2022). Regulating the excited state behaviors of 2-benzooxazol-2-yl-4,6-di-tert-butyl-phenol fluorophore by solvent polarity: A theoretical simulation. Chem. Phys..

[48] Li C.Z., Ma C., Li D.L., Liu Y.F. (2016). Excited state intramolecular proton transfer (ESIPT) of 6-amino-2-(2′-hydroxyphenyl) benzoxazole in dichloromethane and methanol: A TD-DFT quantum chemical study. J. Lumin..

[49] Liu X.M., Wang Y., Wang Y.X., Tao Y.P., Fei X., Tian J., Hou Y.M. (2021). Solvent effect on the excited-state intramolecular double proton transfer of 1,3-bis(2-pyridylimino)-4,7-dihydroxyisoindole. Photochem. Photobiol. Sci..

[50] Guan Y.L., Tang Z., Ju L.P., Zhao J.F. (2022). Solvent polarity-dependent ESIPT behavior for 5-(benzothiazole-2-yl)-4-hydroxyisophthalaldehyde fluorophore: A theoretical study. J. Chin. Chem. Soc..

[51] Yang Y., Liu Y., Yang D., Li H., Jiang K., Sun J. (2015). Photoinduced excited state intramolecular proton transfer and spectral behaviors of aloesaponarin 1. Spectrochim. Acta Part A Mol. Biomol. Spectrosc..

[52] Fleming I. (2011). Molecular Orbitals and Organic Chemical Reactions.

[53] Zhao J.F., Jin B., Tang Z. (2022). Solvent-polarity-dependent conformation and ESIPT behaviors for 2-(benzimidazol-2-yl)-3-hydroxychromone: A novel dynamical mechanism. Phys. Chem. Chem. Phys..

[54] Zhao J.F., Li Z.J., Jin B. (2021). Uncovering photo-induced hydrogen bonding interaction and proton transfer mechanism for the novel salicylaldehyde azine derivative with para-position electrophilic cyano group. J. Lumin..

[55] Frisch M.J., Trucks G.W., Schlegel H.B., Scuseria G.E., Robb M.A., Cheeseman J.R., Scalmani G., Barone V., Mennucci B., Petersson G.A. (2009). Gaussian 09, revision B. 02.

[56] Miertuš S., Scrocco E., Tomasi J. (1981). Electrostatic interaction of a solute with a continuum: A direct utilizaion of AB initio molecular potentials for the prevision of solvent effects. Chem. Phys..

[57] Contreras-Garcia J., Johnson E.R., Keinan S., Chaudret R., Piquemal J.P., Beratan D.N., Yang W. (2011). NCIPLOT: A program for plotting non-covalent interaction regions. J. Chem. Theory Comput..

[58] Lu T., Chen F. (2012). Multiwfn: A multifunctional wavefunction analyzer. J. Comput. Chem..

[59] Liu Z., Lu T., Chen Q. (2020). An sp-hybridized all-carboatomic ring, cyclo 18 carbon: Electronic structure, electronic spectrum, and optical nonlinearity. Carbon.

[60] Humphrey W., Dalke A., Schulten K. (1996). VMD: Visual molecular dynamics. J. Mol. Graph..

