# Supplementary material for: Modulating the ESIPT Mechanism and Luminescence Characteristics of Two Reversible Fluorescent Probes by Solvent Polarity: A Novel Perspective
Source: Molecules. 2024 Apr 5;29(7):1629. doi: 10.3390/molecules29071629 (PMC11013693; doi:10.3390/molecules29071629)
Supplement: Supplementary file 1 [file molecules-29-01629-s001.zip › molecules-2927624-supplementary.pdf]

<u>**Supplementary Material**</u>

Modulating the ESIPT Mechanism and Luminescence Characteristics of Two Reversible Fluorescent Probes by Solvent Polarity: A Novel Perspective

Yang Wang, Hongyan Mu, Yuhang Sun, Jiaan Gao, Xiaodong Zhu and Hui Li *

Jilin Key Laboratory of Solid-State Laser Technology and Application, School of Physics, Changchun University of Science and Technology, Changchun 130022, China; wy318321@163.com (Y.W.); xuemuafm@163.com (H.M.); 13136853259@163.com (Y.S.); gja13622147194@163.com (J.G.); zhu1013288433@163.com (X.Z.)

* Correspondence: huili@cust.edu.cn; Tel./Fax: +86-431-85582465

Table S1. Bond length (Å), bond angles and dihedral angles (°) of HTP-1 and HTP-2 in different electron states.

			O_1-H_1	N_1-H_1	δ(O_1-H_1-N_1)	C_5-C_1-C_2-C_3	C_1-C_2-C_3-C_4	C_2-C_1-C_5-C_6	C_2-C_1-C_7-C_8	C_1-C_2-C_9-C_{10}
HTP-1	ACN	S_0	0.997	1.707	147.79	12.35	48.40	47.66	47.34	47.62
		S_1	0.997	1.709	148.12	58.67	17.02	21.07	24.06	28.03
	ACE	S_0	0.997	1.708	147.74	12.34	48.37	47.64	47.28	47.56
		S_1	0.997	1.711	148.07	53.66	18.37	21.59	25.17	27.97
	DCM	S_0	0.997	1.711	147.63	12.32	48.30	47.58	47.09	47.41
		S_1	0.996	1.714	147.94	52.75	18.63	21.63	25.33	27.85
	n-Hexane	S_0	0.995	1.726	147.08	12.24	48.15	47.13	46.60	46.89
		S_1	0.994	1.727	147.34	50.21	19.12	21.15	25.86	27.45
HTP-2	ACN	S_0	0.990	1.775	145.68	12.56	47.76	47.54	47.31	47.53
		S_1	0.999	1.687	146.60	20.22	38.45	40.82	40.72	43.38
		S_1’	1.881	1.026	126.13	12.63	48.98	48.54	46.39	46.40
	ACE	S_0	0.990	1.776	145.64	12.53	47.82	47.45	47.26	47.49
		S_1	1.000	1.685	146.63	20.24	38.42	40.79	40.65	43.29
		S_1’	2.010	1.022	120.44	9.69	50.46	47.09	47.22	46.04
	DCM	S_0	0.989	1.778	145.54	12.46	47.92	47.26	47.12	47.41
		S_1	1.000	1.682	146.71	20.32	38.35	40.66	40.48	43.10
		S_1’	1.981	1.023	121.90	9.63	50.93	47.14	46.96	45.74
	n-Hexane	S_0	0.988	1.789	145.10	12.23	47.75	46.87	46.63	47.29
		S_1	1.003	1.660	147.13	20.75	38.29	40.16	39.97	42.35
		S_1’	1.886	1.027	126.50	9.90	52.01	47.31	45.92	44.83

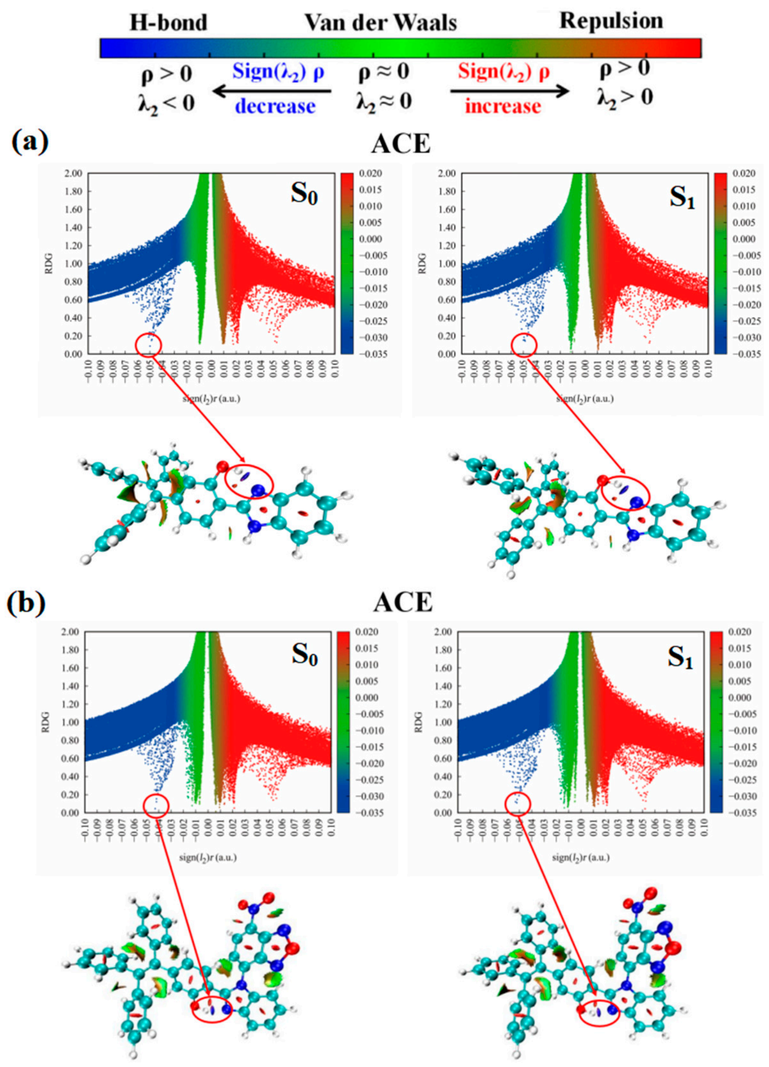

Figure S1. Reduced density gradient (RDG) versus X(r) scatter plots in ACE: (a) HTP-1, (b) HTP-2.

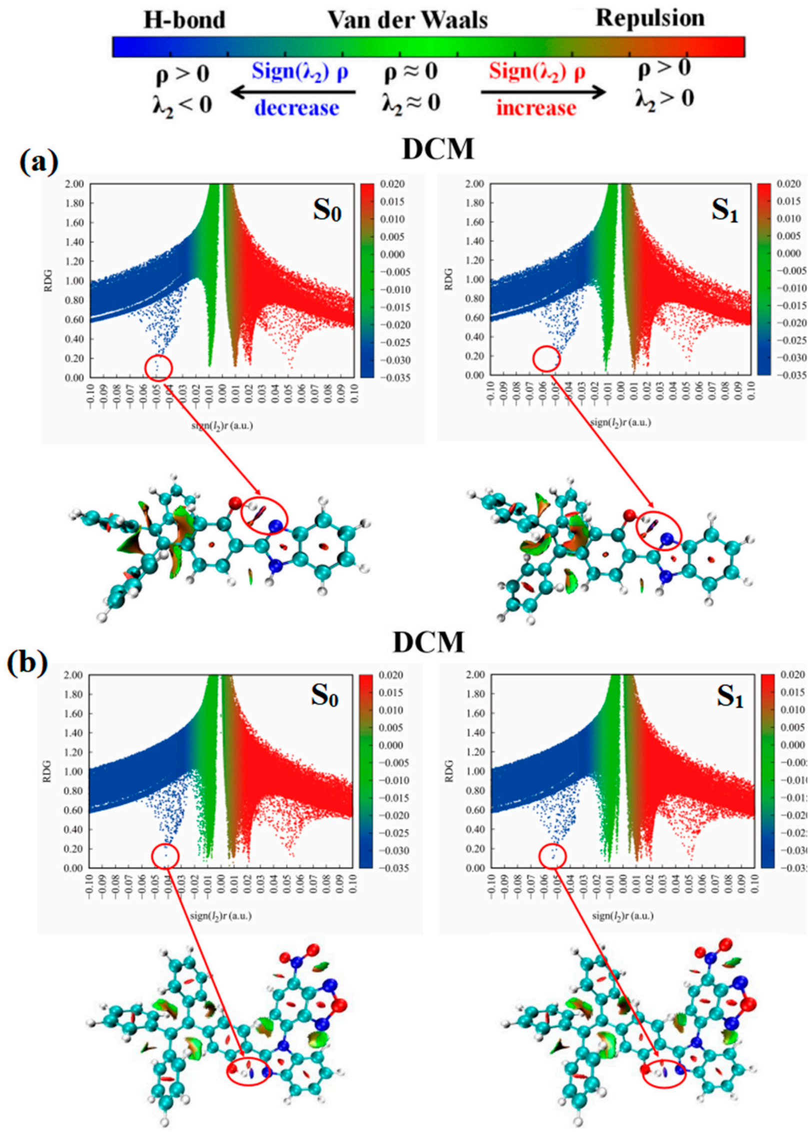

Figure S2. Reduced density gradient (RDG) versus X(r) scatter plots in DCM: (a) HTP-1, (b) HTP-2.

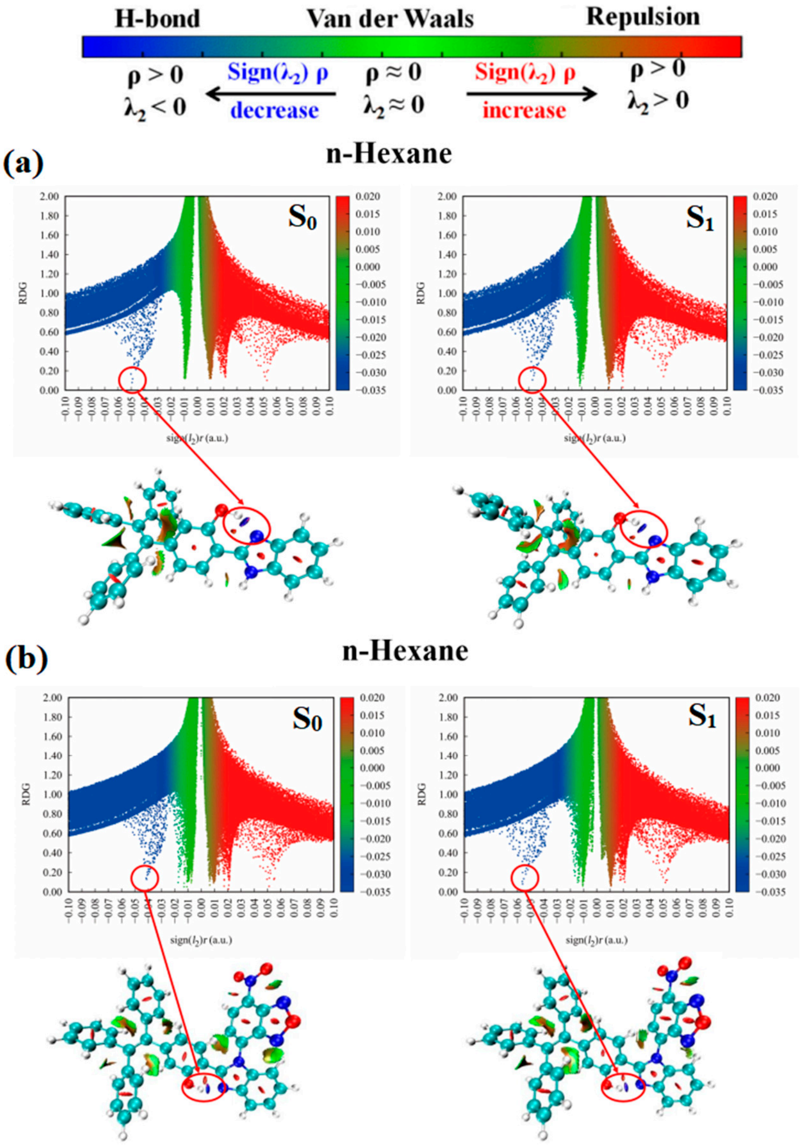

Figure S3. Reduced density gradient (RDG) versus X(r) scatter plots in n-Hexane: (a) HTP-1, (b) HTP-2.

Table S2. Dielectric constant and polarity of four solvents.

Solvents	Dielectric constant	Polarity
ACN	37.5	6.2
ACE	20.7	5.4
DCM	9.08	3.4
n-Hexane	1.58	0.06

Table S3. The experimental and theoretical data of absorption and emission spectra of HTP-1 and HTP-2 in four solvents.

		Exp.	Theor.
HTP-1	ACN	348	351
	DCM	360	352
	n-Hexane	345	352
